# Secretin

**Published:** 1908-03-14

**Authors:** 


### Points in Pathology.

### SECRETIN.

It is well known that each phase of digestion is influenced by that which precedes it; the mastication of food and the secretion of saliva in some way cause an increased secretion of gastric juice; the discharge of chyle through the pylorus causes an increased secretion of pancreatic juice; and so forth. The mechanism by which the secretion of pancreatic juice is thus influenced has been the subject of much research work of recent years, notably at the hands of Professor Starling.

### Physiology.

The outcome of these researches is briefly as follows:—

The duodenal mucosa has two very important functions besides those of resorption. It has a special external secretion, which it pours into the lumen of the bowel; and it has a special internal secretion, which it returns to the blood-stream. The name secretin has been given to the substance which, produced by the duodenum, is returned to the blood-stream; and its function is to stimulate the cells of the pancreas to increased activity. The other duodenal secretion has as yet no generally accepted name, but it has a very important function. The pancreatic juice, as it leaves the pancreatic cells and lies in the ducts of the gland, is comparatively inert, its ferments being incompletely active; but when they mix with the duodenal secretion the pancreatic ferments are at once matured and made perfect for digestive purposes. The importance of this is manifest. It explains why the pancreas and its ducts are not being constantly auto-digested, and it may perhaps explain why they sometimes do become auto-digested; if, for example, some irregularity of peristalsis, the result of injury, or of a calculus at the ampulla of Vater, or something of that sort, were to cause some of the duodenal juices to regurgitate into the pancreatic ducts, the ferments in the juices therein would be converted from an inactive to a highly active condition, and the result would be some degree of pancreatic digestion associated with inflammation—in other words, acute pancreatitis, hæmorrhagic or otherwise, with fat necrosis and peritonitis.

The secretin, on the other hand, is not concerned with activating the pancreatic ferments; its function is to cause increased secretion of normal pancreatic juice at the right time. Secretin can be prepared by suitably extracting duodenal tissue; when administered to animals it causes an enormous increase in pancreatic activity, and unless the dose is very carefully regulated the intestinal mucosa may become tremendously irritated and inflamed as the result.

### Therapeutic Possibilities.

It is not as yet very safe to employ secretin itself in the treatment of diseases in man. More experiments upon animals are required before all its possible dangers are fully understood; but the knowledge of secretin and of its effects upon the pancreas opens up a prospect of some day being able to combat successfully such diseases as are due to pancreatic insufficiency. Diabetes mellitus occurs to one at once. We have no certain knowledge that diabetes is really due to trouble in the pancreas; there is much evidence to show that it often has nothing to do with the pancreas at all; but if there are even only some cases in which a pancreatic lesion is at the root of the diabetes, secretin might well be of service in its treatment. It has been tried already, but hitherto without much benefit; this, however, does not show that it will never be of use in this affection; many more experiments are required, both upon secretin itself, and still more upon diabetes, in order to settle if possible the exact rôle, if any, of the pancreas in the pathogeny of the latter.

If the time is not yet ripe for the use of secretin in the treatment of disease, much advantage may already be got from a clear understanding of the factors which modify the two secretions of the duodenum—secretin on the one hand, and the activating substance secreted into the contents of the bowel on the other. Both these secretions are called forth in abundance whenever the acid products of gastric digestion pass the pylorus and enter the first part of the duodenum. It is the *acid* which calls forth the duodenal secretions; they may be evoked experimentally by applying any ordinary weak solution of acid to the mucosa. Supposing, therefore, that no acid products of digestion reached the duodenum, the actual amount of pancreatic juice secreted would be deficient, and at the same time the activation of the ferments in the juice would be incomplete. The result would be inefficiency in digestion. It does not follow, of course, that the patient would lose weight, or even be dangerously ill; for fortunately the human frame has an immense amount of adaptibility, and there are very wide margins between which the activity of secretions and absorptions may vary without there being any actual danger; but when the pancreatic juice is deficient, nature will revolt against giving it more work to do than it can accomplish, and loss of appetite will be one of the earliest results. The administration of acids by the mouth will tend to correct this, and it is interesting to know this physiological *raison d'être* of the many efficacious acid remedies for dyspepsia; it is very probable that they do not act so much by assisting the activity of the pepsin as they do by causing the chyle, as it enters the duodenum, to be sufficiently acid to evoke a proper formation of secretin. This being so, it is not at all surprising that the acid mixtures often do more good when they are given half an hour after meals than when they are given before eating.

Again, it has long been known that deficiency of the HCl in the gastric juice is by no means restricted to cases of gastric carcinoma; there is similar deficiency or absence of free HCl in many other conditions, such as malignant disease elsewhere than in the stomach, cirrhosis of the liver in its later stages, fevers of all kinds, chronic heart disease with failing compensation, chronic Bright's disease sometimes, phthisis, and so forth. It is very possible—nay, probable—that the cachexia associated with many of these diseases is at least accentuated by the absence of proper pancreatic secretion owing to lack of sufficient acid-stimulation of the duodenum; it is clear that, if this be so, the administration of an acid mixture should be indicated, and that the best time to give the acid would be when the gastric contents have become sufficiently disintegrated to be passing at their maximum rate through the pylorus into the duodenum—a time which varies with the motility of the stomach and with the nature of the last meal eaten. It would seem wise, moreover, to give more than a single dose of the acid mixture after each meal; in some cases the acid would do most good if given in three or more small doses, at intervals of half an hour, to ensure each portion of the gastric contents containing acid when it reached the duodenum. It is clear, moreover, that if one would be accurate in treatment, any opportunity that may be afforded for estimating the acidity of the gastric juice upon varying diets and at varying intervals after meals should always be taken advantage of. Lavage can scarcely be undertaken for this purpose, except in a few cases; but when lavage is not possible occasional vomits may assist greatly; no vomit should be thrown away until it has been examined for acid, and the result interpreted in relation to the diet eaten and the time that has elapsed since the last meal, as we have already discussed elsewhere. The importance of secretin and the physiology of the duodenum in relation to the pancreas is also great in connection with gastro-jejunostomy, affording an additional argument against the performance of this operation upon any but essential grounds. As a matter of fact, however, the passage of acid gastric contents into the jejunum appears to be a better alternative to their passage into the duodenum than might have been expected. Researches have shown that resorption of fat and of proteid is by no means very deficient after gastro-jejunostomy, particularly if a portion of the gastric contents can still find their way viâ the pylorus. Experiments may some day show that the jejunum as well as the duodenum has some power of producing secretin.

Be this as it may, enough has been said to indicate the important advances that have recently been made in connection with the chemistry of the functional relationship between the duodenum and the pancreas, and to outline the important points in treatment upon which light has been thrown by researches into the physiology and pathology of that internal secretion of the duodenum which is known as secretin.

